# The complete chloroplast genome of *Bletilla ochracea* Schltr., a medicinal plant with yellow flowers

**DOI:** 10.1080/23802359.2021.1992316

**Published:** 2022-06-10

**Authors:** Dan Zhao, Zhishang Liu, Tao Zhou, Zhikun Wu, Yongping Zhang, Chenghong Xiao

**Affiliations:** a Guizhou University of Traditional Chinese Medicine, Guiyang, China; b Administration center of Nonggang National Nature Reserves, Longzhou, China

**Keywords:** *Bletilla ochracea*, Chloroplast genome, phylogenetic analysis

## Abstract

*Bletilla ochracea* Schltr. (Orchidaceae) is a traditional medicinal plant widely distributed in the south-central part of China. The complete chloroplast genome of *B. ochracea* was sequenced using the Illumina HiSeq X Ten platform. The chloroplast genome was 160,018 bp in length, which contained two short inverted repeat (IRa and IRb) regions of 26,295 bp and was separated by a large single copy (LSC) region of 88,270 bp and a small single copy (SSC) region of 19,158 bp. The GC content of the whole chloroplast genome was 37.2%. The chloroplast DNA of *B. ochracea* consisted of 114 distinct genes, including 80 protein-coding genes, 4 ribosomal RNA genes, and 30 transfer RNA genes. The phylogenetic tree showed that *B. ochracea* was sister to *B. formosana*. Meanwhile, the monophyletic clade formed by all species of genus *Bletilla* was closely related to genus *Thunia*.

*Bletilla ochracea* Schltr., the only yellow-flowered member of the genus *Bletilla*, has a wide distribution in the south-central regions of China such as Guangxi, Sichuan, Yunnan, etc. The height of the whole plant is 25–55 cm, and the rhizome is compressed or irregularly shaped. In China, the roots and rhizomes of this plant are used for medicinal purposes, such as for reducing swelling and stopping bleeding in the lungs and stomach (Wu et al. 2009). Owing to its wide distribution and several medical uses, it is necessary to sort out the background of the evolutionary biology of *B. ochracea* for further research, with the aim of supporting the advancement of natural medicine. Meanwhile, the chloroplast genomes are valuable data for phylogenetic analysis, genetic diversity evaluation, and plant molecular identification (Sun et al. 2020; Wang et al. 2021). In the present study, we reported the complete chloroplast genome of *B. ochracea* based on Illumina sequencing data (GenBank accession number: MZ221775), which would be helpful for its phylogenetic analysis and further utilization for various purposes.

Fresh *B. ochracea* samples were collected from Longzhou County, Guangxi Province, China (22°20′21″N, 106°51′14″E). We extracted the total genomic DNA from the fresh leaves of a single individual plant using the Plant DNA Kit (D200-200, http://www.gene-better.cn) from GeneBetter Life Science Co., Ltd. and purified it using a Wizard DNA cleanup system (Promega, Madison, WI, USA). The specimen and extracted DNA were deposited at Guizhou University of Traditional Chinese Medicine (contact person: Dan Zhao; email: zhaodan8964@126.com) under the voucher number HHBJLZ02. A paired-end library was constructed using the NEBNext Ultra^TM^ DNA Library Prep Kit. Paired-end (150 bp) sequencing was performed by Novogene Bioinformatics Technology Co., Ltd. (Beijing, China), using the Illumina HiSeq X-Ten platform. The Next-generation Sequencing QC Toolkit was used for quality control and for filtering low-quality reads. The chloroplast genome was assembled by the program GetOrganelle with the default reference database (Jin et al. 2020). Gene annotation of *B. ochracea* was performed using CPGAVAS2 annotation (Shi et al. 2019). Where necessary, the positions of start and stop codons and boundaries between introns and exons were manually corrected.

The chloroplast genome of *B. ochracea* was 160,018 bp in length, which contained two short inverted repeat (IRa and IRb) regions of 26,295 bp and was separated by a large single copy (LSC) region of 88,270 bp and a small single copy (SSC) region of 19,158 bp. The GC content of the whole chloroplast genome was 37.2%. The cpDNA of *B. ochracea* consisted of 114 distinct genes, including 80 protein-coding genes, 4 ribosomal RNA genes, and 30 transfer RNA genes. Of these, 19 genes were duplicated in the IR regions and 19 genes contained either one or two introns. A total of 17 genes contained a single intron, whereas 2 (*ycf3* and *clpP*) contained double introns. The *rps12* gene was a trans-spliced gene with the 5′ end located in the LSC region and the 3′ end located in the IR region. The gene *trnK-UUU* had the largest intron, which contained the *matK* gene.

To confirm the phylogenetic position of *B. ochracea*, a total of 36 complete chloroplast genomes of Epidendreae were obtained from GenBank and genus *vanda* was regarded as an outgroup (Figure 1). A phylogenetic IQ-tree was constructed using PhyloSuite under the GTR + F + I + G4 model with 1000 bootstrap replicates (Nguyen et al. 2015; Zhang et al. 2020). The phylogenetic tree showed that *B. ochracea* was sister to *B. formosana*. Meanwhile, the monophyletic clade formed by all species of genus *Bletilla* was closely related to genus *Thunia*. The chloroplast genome of *B. ochracea* may provide genetic information for future research on the conservation and taxonomy of species in the family Orchidaceae.

**Figure 1.**
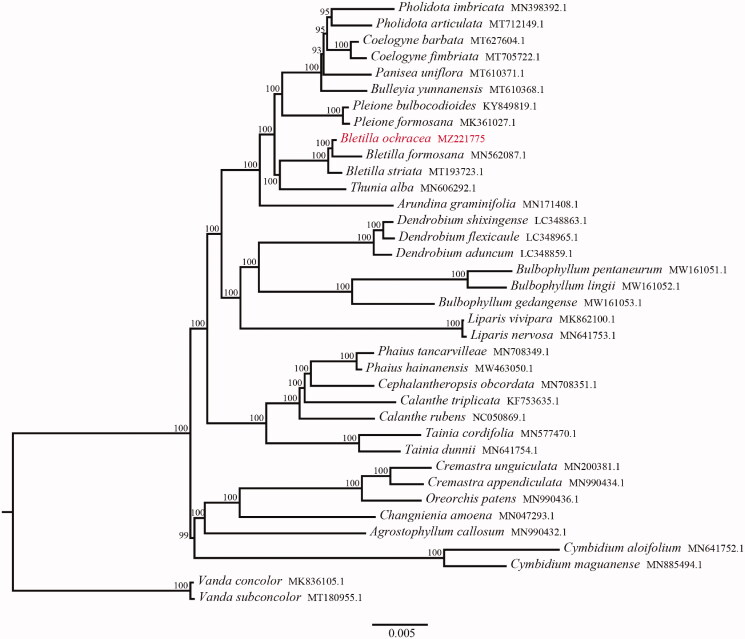
Phylogenetic tree reconstruction of 37 taxa using maximum likelihood (ML) methods in the chloroplast genome sequences. An ML bootstrap support value is presented at each node.

## Data Availability

The genome sequence data that support the findings of this study are openly available in GenBank of NCBI at (https://www.ncbi.nlm.nih.gov/) under the accession no.MZ221775. The associated BioProject, SRA, and Bio-Sample numbers are PRJNA730110, SRR14554259, and SAMN19217945 respectively.
